# The Role of Mast Cells in the Defence against Pathogens

**DOI:** 10.1371/journal.ppat.1002619

**Published:** 2012-04-26

**Authors:** Mirjam Urb, Donald C. Sheppard

**Affiliations:** 1 Department of Microbiology and Immunology, McGill University, Montréal, Québec, Canada; 2 Department of Medicine, McGill University, Montréal, Québec, Canada; Duke University Medical Center, United States of America

Although mast cells are best known for their role in mediating allergic diseases, recent studies have highlighted the important role that these cells play in the protection against infection with a variety of organisms.

## What Are Mast Cells?

Mast cells are leukocytes that are derived from haematopoietic progenitor cells. They circulate in the blood in an immature form before migrating to vascularised tissues, where they undergo final differentiation and maturation with the help of stem-cell factor and other cytokines secreted by endothelial cells and fibroblasts. Mast cells are found in most tissues of the body, particularly in locations that are in close contact with the external environment, such as skin, airways, and intestines. They are, therefore, ideally placed to participate in the early recognition of pathogens. Activation of mast cells results in the release of a variety of soluble factors. Within seconds of stimulation, mast cells can undergo degranulation, rapidly releasing pre-formed mediators present within cytoplasmic granules, including histamine, the proteases tryptase and chymase, and pre-formed tumour necrosis factor-alpha (TNF-α; reviewed in [1], [2]). Shortly after the initiation of degranulation, mast cells can produce lipid-derived eicosanoids such as prostaglandin D_2_ and leukotriene C_4_ (LTC_4_). Finally, over the course of hours, the transcriptional up-regulation of cytokines and chemokines, including TNF-α and interleukin-4, can be observed. Importantly, each of these responses may occur alone or in combination depending on the stimulus. Because of their location, their plasticity, and the various mediators they produce, mast cells are important immune effector and modulatory cells that help link innate and adaptive immunity in the fight against pathogens.

## How Do Pathogens Activate Mast Cells?

The best studied mechanism for the activation of mast cells is via stimulation of the high-affinity immunoglobulin E (IgE) receptor FcεRI (reviewed in [3]). Binding of an antigen by FcεRI-bound specific IgE leads to FcεRI clustering, which in turn induces downstream signalling events and ultimately the release of mediators. Although initially described in the context of allergy, this response is important in the response to parasites, including nematodes and malaria. Mast cells also express Fc receptors that bind IgG and a variety of complement receptors, and therefore can potentially respond to opsonised organisms. The role of these receptors in mast cell activation during infection remains less well defined.

As with other leukocytes, mast cells can also be activated by directly interacting with pathogens through pattern recognition receptors (PRRs), including the Toll-like receptors (TLRs), Nod-like receptors, C-type lectins such as Dectin-1, and the glycosylphosphatidylinositol-anchored protein CD48. Selective engagement of PRRs is also an important mechanism in governing the type of mast cell response. For example, while peptidoglycan stimulation of bone marrow-derived mast cells via TLR2 leads to both cytokine release and degranulation, lipopolysaccharide (LPS) stimulation through TLR4 results in cytokine release alone [4]. Furthermore, Dectin-1 binding of fungal β-glucan induces the release of LTC_4_ by mast cells [5] while CD48 binds to the *Escherichia coli* adhesin FimH, and induces the release of TNF-α [6].

## How Do Mast Cells Contribute to Host Defence?

**Figure 1:**
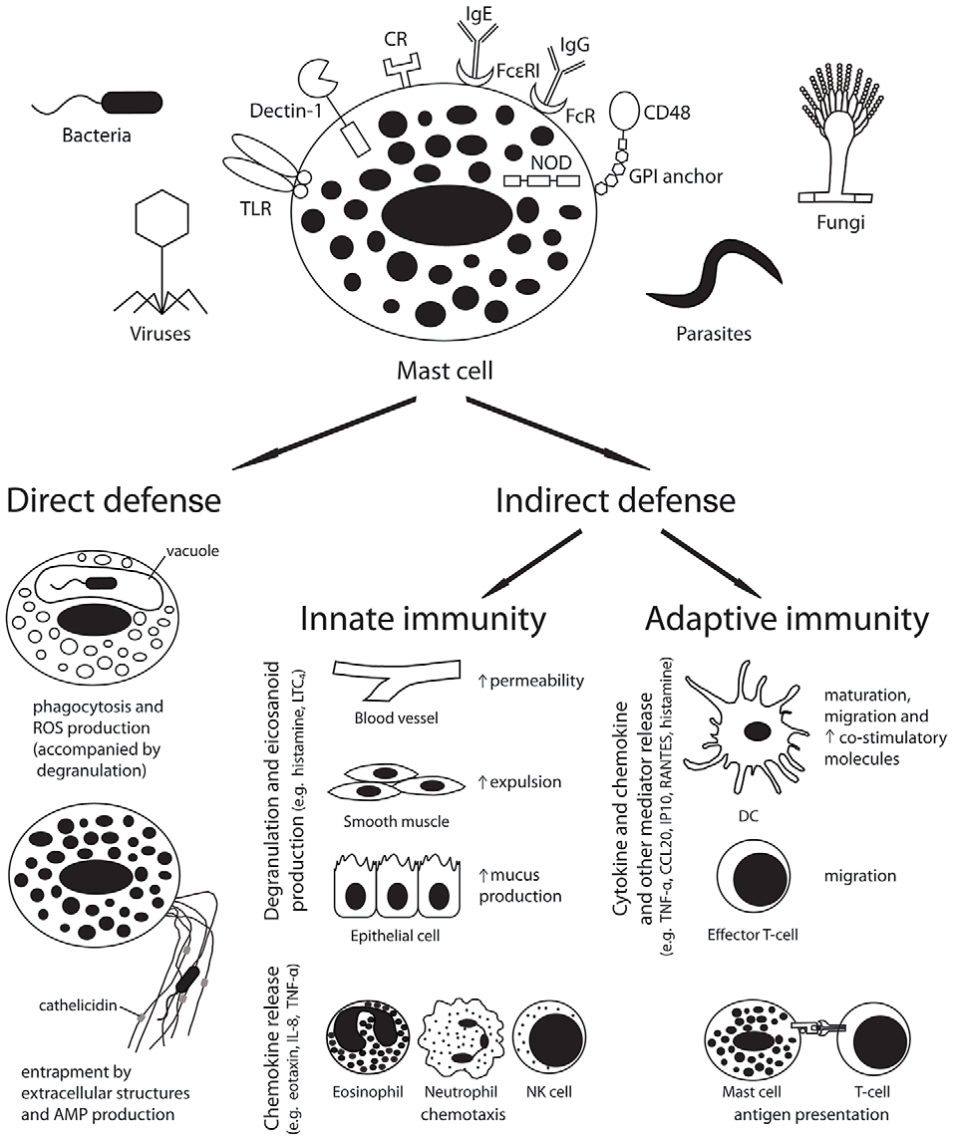
Mast cells play a pivotal role in the host defence against pathogens. Pathogen recognition by host receptors leads to mast cell activation and both direct and indirect antimicrobial responses. Abbreviations: AMP, antimicrobial peptide; CCL20, chemokine (C-C motif) ligand 20; CR, complement receptor; DC, dendritic cell; FcR, Fc receptor; GPI, glycosylphosphatidylinositol; Ig, immunoglobulin; IL-8, interleukin-8; IP10, interferon gamma-induced protein 10; LTC4, leukotriene C4; NK cell, natural killer cell; NOD, nucleotide oligomerisation domain like receptor; ROS, reactive oxygen species; TLR, toll-like receptor; TNF-α, tumour necrosis factor-alpha.

Mast cells are well placed to serve as immune sentinel cells to both respond directly to pathogens and send signals to other tissues to modulate both innate and adaptive immune responses (Figure 1).

Mast cells can participate in direct killing of organisms by phagocytosis and reactive oxygen species production [7], and can produce antimicrobial peptides, such as cathelicidins, both constitutively and in response to LPS or lipoteichoic acid exposure [8]. These peptides were found to mediate killing of Group A streptococci (GAS) in vitro and in vivo [8]. Additionally, similar to neutrophils, mast cells have been found to produce extracellular traps that encompass and kill organisms, such as GAS, in vitro [9]. Although these microbicidal responses may be important in some infections, the relatively small number of mast cells in tissues suggests that indirect effects of mast cells in coordinating host innate and adaptive responses may be more important in the balance of host defence. Future studies defining the relative contributions of direct and indirect antimicrobial effects are required.

Mast cells can modulate host innate immune responses through the release of granular and secreted mediators (reviewed in [1], [2]). The release of histamine and other vasoactive mediators increases vascular permeability and local blood flow, and can act on smooth muscle to increase the expulsion of mucosal parasites. In addition, histamine enhances epithelial cell mucus production, which may aid in pathogen immobilisation and cytoprotection. Finally, mast cell production of chemotactic factors can enhance the recruitment of multiple inflammatory cells including eosinophils (eotaxin), natural killer (NK) cells (IL-8), and neutrophils (IL-8 and TNF-α).

Mast cell products have also been implicated in the regulation of adaptive immune responses (reviewed in [1], [2]). Mast cell–derived cytokines and chemokines can enhance the migration of dendritic cells (DCs; TNF-α and CCL20) and effector T cells (CXCL10/IP10 and CCL5/RANTES) to the site of infection and to draining lymph nodes. Mast cells can also function directly as antigen-presenting cells, particularly for CD8^+^ T cells. In addition, mast cell products can enhance the maturation of immature DCs, and up-regulate antigen presentation and the expression of co-stimulatory molecules. Interestingly, while mast cell–derived histamine was observed to favour the polarization of naive T cells towards a Th2 phenotype by reducing DC production of IL-12 and increasing IL-10 secretion in response to LPS [10], direct contact with mast cells can prime DCs to promote Th17 and Th1 polarization in vitro [11]. Although these findings require confirmation in vivo, they suggest that mast cells may act to promote the development of different immune responses depending on environmental and other cues.

Importantly, while mast cell responses may act to increase host defence locally at the site of infection, it is also possible that mast cell–mediated enhancement of inflammation could induce damage of host tissues and worsen outcome during some infections. In support of this hypothesis, a recent study observed that while intraperitoneal mast cells were found to be protective in a model of experimental polymicrobial intra-abdominal sepsis, extraperitoneal mast cells in the same system produced pro-inflammatory IL-6, which was associated with increased mortality [12]. This increase in mortality was associated with increased circulating histamine, suggesting that severe sepsis led to the induction of systemic mast cell degranulation distal to the site of infection, and subsequent overproduction of pro-inflammatory mediators.

## Mast Cells Have Been Implicated in the Defence against Which Pathogens?

Recent studies have demonstrated that mast cells play a protective role against many pathogens. Significant advances have been made using mast cell–deficient mice, although the specific mechanisms by which mast cells inhibit most pathogens remain relatively undefined.

The first evidence supporting the protective role of mast cells against pathogens came from studies of parasitic infections including helminths, nematodes, and protozoa (reviewed in [1], [2]). Experiments using mast cell–deficient mice have found that mast cells accelerate hookworm expulsion from the gut in association with the production of mast cell protease 2. Similar studies in models of *Trichinella spiralis* and *Strongyloides* infection have found that mast cells mediate gut expulsion of nematodes and limit the parasite tissue burden. Moreover, mast cell–deficient mice develop increased parasite burden and larger lesions during infection with *Leishmania major* in association with a reduction in inflammation and IL-12 production at the site of infection. Finally, a critical role for mast cell–derived TNF in limiting parasitaemia in a murine model of malaria has been demonstrated by reconstituting mast cell–deficient mice with mast cells derived from wild-type and TNF-deficient mice [13].

More recently, the contributions of mast cells to antibacterial immunity have also been established, particularly with respect to gram-negative bacteria (reviewed in [1], [2]). Mast cells have been found to attenuate experimental pulmonary infection with *Klebsiella pneumoniae* [14] and *Mycoplasma pneumoniae*; *Pseudomonas aeruginosa* and GAS skin infection; *Haemophilus influenzae* otitis media; and *E. coli* peritoneal and urinary infections, as well as polymicrobial intra-abdominal sepsis [15].

Evidence that mast cells mediate antiviral immunity is more limited. Mast cell activation by synthetic viral dsRNA led to the recruitment of CD8^+^ T cells to the site of infection that was absent in mast cell–deficient mice [16]. Dengue infected mast cell–deficient mice had an increased viral burden within draining lymph nodes due to the lack of recruitment of NK and NK T cells to the site of infection [17]. Conversely, however, in HIV infection, mast cells may serve as a viral reservoir during latent infection [18].

The role of mast cells in the pathogenesis of fungal infection is even less well understood. In vitro studies have found that mast cells released LTC_4_ in response to zymosan, a *Saccharomyces cerevisiae* cell wall preparation [5]. A single study examining the interaction of live fungi and mast cells in vitro found that *Aspergillus fumigatus* hyphae induced degranulation of mast cells via an IgE-independent mechanism [19]. Extending these studies in vivo will be critical for understanding the role of mast cells in fungal infections, as there may be important differences between the role of mast cells in the defence against fungi and other eukaryotic pathogens such as parasites. For example, while the induction of a mast cell–associated Th2 response is classically protective in parasitic infection, a Th2 response is usually detrimental during fungal infection [20].

## Could Enhancing Mast Cell Function Protect against Infection?

In allergic diseases, mast cells are seen as harmful triggers of chronic inflammation, and mast cell stabilizing agents and inhibitors are frequently used as treatment. However, emerging data suggest that mast cells are crucial in protecting the host from many infections. Although substantial effort has been directed towards defining and reversing the effects of corticosteroids and other immunosuppressive agents on neutrophil, macrophage, and dendritic cell function, similar studies are lacking for mast cells. Failure of mast cells to function as immune sentinels early in infection may play an important role in mediating susceptibility to infection in patients receiving corticosteroids or other mast cell–suppressing agents. Future studies will be required to understand the effects of these agents on specific aspects of mast cell function and subsequent susceptibility to specific infections. New strategies focused on enhancing the beneficial roles of mast cells may facilitate the early response to pathogens when the microbial burden is low.
